# Reproducibility of Fat_max_ and Fat Oxidation Rates during Exercise in Recreationally Trained Males

**DOI:** 10.1371/journal.pone.0097930

**Published:** 2014-06-02

**Authors:** Ilaria Croci, Fabio Borrani, Nuala Byrne, Rachel Wood, Ingrid Hickman, Xavier Chenevière, Davide Malatesta

**Affiliations:** 1 The University of Queensland Diamantina Institute, The University of Queensland, Translational Research Institute, Brisbane, Australia; 2 School of Human Movement Studies, University of Queensland, Brisbane, Australia; 3 Institute of Sport Sciences University of Lausanne, University of Lausanne, Lausanne, Switzerland; 4 Department of Physiology, University of Lausanne, Lausanne, Switzerland; 5 Institute of Health and Biomedical Innovation, Queensland University of Technology, Brisbane, Australia; 6 Bond Institute of Health and Sport, Bond University, Robina, Australia; 7 Department of Nutrition and Dietetics, Princess Alexandra Hospital, Brisbane, Australia; 8 Mater Medical Research Institute, Mater Mother’s Hospital, Brisbane, Australia; 9 Department of Medicine, University of Fribourg, Fribourg, Switzerland; University of Sao Paulo, Brazil

## Abstract

Aerobic exercise training performed at the intensity eliciting maximal fat oxidation (Fat_max_) has been shown to improve the metabolic profile of obese patients. However, limited information is available on the reproducibility of Fat_max_ and related physiological measures. The aim of this study was to assess the intra-individual variability of: a) Fat_max_ measurements determined using three different data analysis approaches and b) fat and carbohydrate oxidation rates at rest and at each stage of an individualized graded test. Fifteen healthy males [body mass index 23.1±0.6 kg/m^2^, maximal oxygen consumption (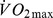) 52.0±2.0 ml/kg/min] completed a maximal test and two identical submaximal incremental tests on ergocycle (30-min rest followed by 5-min stages with increments of 7.5% of the maximal power output). Fat and carbohydrate oxidation rates were determined using indirect calorimetry. Fat_max_ was determined with three approaches: the sine model (SIN), measured values (MV) and 3^rd^ polynomial curve (P3). Intra-individual coefficients of variation (CVs) and limits of agreement were calculated. CV for Fat_max_ determined with SIN was 16.4% and tended to be lower than with P3 and MV (18.6% and 20.8%, respectively). Limits of agreement for Fat_max_ were −2±27% of 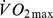 with SIN, −4±32 with P3 and −4±28 with MV. CVs of oxygen uptake, carbon dioxide production and respiratory exchange rate were <10% at rest and <5% during exercise. Conversely, CVs of fat oxidation rates (20% at rest and 24–49% during exercise) and carbohydrate oxidation rates (33.5% at rest, 8.5–12.9% during exercise) were higher. The intra-individual variability of Fat_max_ and fat oxidation rates was high (CV>15%), regardless of the data analysis approach employed. Further research on the determinants of the variability of Fat_max_ and fat oxidation rates is required.

## Introduction

Carbohydrate and fat are the two main sources of energy that sustain oxidative metabolism. Their relative utilization during aerobic exercise depends largely on exercise intensity [1], [2]. The whole-body carbohydrate oxidation rate (CHO_ox_) increases with the workload, whereas the whole-body fat oxidation rate (F_ox_) increases from low to moderate exercise intensities, and then markedly declines at high intensities. The exercise intensity at which the maximal fat oxidation (MFO) rate occurs has been defined as Fat_max_ [3]. Aerobic exercise training performed at Fat_max_ has the potential to increase F_ox_ and insulin sensitivity in obese patients [4] and in individuals with metabolic syndrome [5]. In patients with type 2 diabetes, aerobic training targeted at Fat_max_ was shown to have a greater effect on body composition and glucose control than high intensity interval training [6].

To determine Fat_max_, a submaximal graded exercise test using indirect calorimetry is performed, and data is analyzed with two main steps. First, F_ox_ and CHO_ox_ at each stage of the test are calculated from indirect calorimetry measures [oxygen consumption (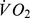) and carbon dioxide production (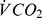)] by means of the stoichiometric equations [7]. Subsequently, F_ox_ values are plotted as a function of exercise intensity and Fat_max_ is identified with one of the following four commonly used methods: a) the determination of the maximal value of measured F_ox_ reached during each stage of the graded exercise test and identification of the corresponding intensity (measured values approach, MV) [3], [8]–[10], b) the construction of a 3^rd^ polynomial fitting curve (P3) [11], c) the Sine model (SIN) [12] and d) the non-protein “respiratory quotient technique” [13].

Knowledge of the reproducibility of Fat_max_ is necessary for establishing its usefulness as a parameter for training prescription and for adequately interpreting outcomes from research studies. To date, there has been limited research into the reproducibility of testing Fat_max_, and findings to date are conflicting and have methodological limitations [8], [13], [14]. Achten *et al.* [8] found a coefficient of variation (CV) for Fat_max_ of 9.6% in 10 endurance athletes tested on three occasions and concluded that Fat_max_ measurements are reliable. Perez-Martin *et al.* [13] tested 10 healthy males on two occasions, reported a CV for Fat_max_ of 11.4% and suggested this to be a “satisfactory result”. Conversely, Meyer *et al.* [14] studied 21 recreationally trained men and women who completed the test twice. The limits of agreement (LoA) for oxygen consumption at Fat_max_ corresponded to a heart rate (HR) difference of 35 bpm between the two tests, which lead them to conclude that the intra-individual variability in Fat_max_ measurements is too large to recommend using this parameter for prescribing exercise training. In addition to coming to different conclusions, these studies had methodological limitations in terms of testing protocol and data analysis approach. Data analysis to determine Fat_max_ was performed using the MV [8], [14] or the “respiratory quotient technique” [13] approaches. However, Chenevière *et al.* [12] recently showed that the employment of a mathematical model (SIN) provides a more complete description of the Fat_ox_ kinetics as a function of exercise intensity and more accurate Fat_max_ measures than the “respiratory quotient technique” approach. Secondly, in two of these studies [8], [14], the starting workload of the graded test occurred on average at ∼45% of the maximal oxygen uptake (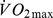), therefore not providing information on substrate metabolism at low intensities, while in the other [13], the protocol included a limited number of exercise stages, therefore limiting information for determining Fat_max_. Furthermore, the statistical approach to assess reliability used by Achten *et al.* and Perez-Martin *et al.* was not comprehensive given that only CVs were reported [15]. Other measures of variability such as the LoA were not calculated.

Crucially, even though Fat_max_ is calculated from F_ox_ values at each stage of a submaximal graded test, the reproducibility of F_ox_ over a wide range of exercise intensities has not been assessed. Some studies have evaluated the intra-individual variability of the physiological parameters used to determine substrate oxidation (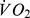,
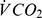
and respiratory exchange ratio or RER). The authors reported that 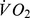 and 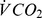 were reliable in resting conditions [16], [17] and that CVs for 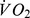,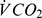 and RER were lower than 5% in response to each stage of an incremental exercise test [8], [18], [19]. However, while CVs of 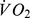,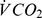 and RER are often reported to inform on the variability in substrate oxidation rates, this might be misleading. The relationship existing between those CVs and the variability of F_ox_ and CHO_ox_ has not been established.

Limited information is available on the reproducibility of Fat_max_ and on the reproducibility of CHO_ox_ and F_ox_ at each stage of a graded test. It was therefore the aim of this study to assess the intra-individual variability of: a) Fat_max_ measurements determined using three different data analysis approaches (SIN, P3 and MV), and b) CHO_ox_ and F_ox_ at rest and in response to each stage of an individualized graded test. A further aim was to investigate how the CVs of 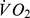,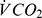 and RER are related to the CV of F_ox_.

## Methods

### Ethics Statement

The study was conducted in accordance with ethical principles of the 1964 World medical Declaration of Helsinki and was approved by the human research ethics committee of the University of Lausanne (Switzerland). All test procedures, risks and benefits associated with the experiment were fully explained, and written informed consent was obtained from all participants.

### Subjects

Fifteen healthy, moderately trained male volunteers (see Table 1 for anthropometric and physical characteristics) were recruited to participate in this study. All participants were of normal weight according to the World Health Organization (Body Mass Index<25 kg⋅m^−2^), non-smokers and disease-free. They were not taking regular medications and were screened for the absence of electrocardiographic abnormalities at rest and during exercise.

**Table 1:** Subject characteristics.

	*n* = 15
Age (years)	27.4±4.0
Height (cm)	180±5
Weight (kg)	74.5±7.6
BMI (kg⋅m^2^)	23.1±2.3
Body fat (%)	14.4±2.9
Fat-free mass (kg)	63.7±5.9
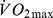 (mL⋅kg^−1^⋅min^−1^)	52.0±7.7
HR_max_ (beats⋅min^−1^)	185±11
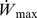 (Watts)	322±51

Values are means ± SD. *n*, number of subjects; BMI, body mass index; 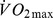, maximal oxygen uptake; HR_max_, maximal heart rate; 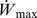, maximal aerobic power output.

### General Design

Each participant completed three test sessions. In the first session anthropometric measurements (*i.e.,* stature, body mass and body composition) were taken and a maximal incremental test on a cycle ergometer was performed. In the remaining two sessions the subjects performed an identical submaximal incremental test (Test 1 and Test 2). The two tests were performed in the morning (start of exercise between 7 and 8 am) after a10-hour overnight fast. They were separated by 3 to 7 days and performed at the same time of day to avoid circadian variance. The volunteers were asked to fill in a 1-day food diary on the day before Test 1 and to repeat this diet before Test 2. Furthermore, participants were asked to refrain from vigorous exercise and alcohol and caffeine consumption in the 24 hours prior to testing. Participants were familiarized with the equipment prior to testing.

### Anthropometric Measurements

Body composition (fat mass and percentage of body fat) was estimated from skin-fold thickness measurements at four sites according to the methods of Durnin and Womersley [20].

### Maximal Exercise Test

A maximal incremental test on a cycle ergometer (Ebike Basic BPlus, General Electric, Niskayuna, NY, USA) to determine maximal oxygen uptake (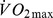) and maximal aerobic power output (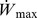) was performed. After a 5-min rest period and a 5-min warm-up at 60 W, output was increased by 30 W every minute until volitional exhaustion. 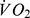
was considered as maximal when at least three of the following four criteria were met [21]: 1) a plateauing of 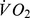 (defined as an increase of no more than 2 mL⋅kg^−1^⋅min^−1^ with an increase in workload) during the latter stages of the exercise test, 2) an HR>90% of the age-predicted maximum (220-age), 3) an RER>1.1 and 4) an inability to maintain the minimal required pedaling frequency (*i.e.* 60 rpm) despite maximum effort and verbal encouragement. 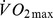
was calculated as the average 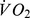 over the last 20 seconds of the last stage of the test.

### Submaximal Graded Exercise Tests (Test 1 and Test 2)

Test 1 and Test 2 were characterized by two phases: a pre-exercise resting phase (rest) and a submaximal incremental exercise test. They were carried out under identical circumstances with an identical protocol. Data from these two tests were subsequently employed for reliability calculations.

In the pre-exercise resting phase (rest), participants were seated for 30-min on the cycle ergometer and respiratory measures were collected during the last 15-min of this sitting period. Subsequently, a submaximal incremental exercise test to determine whole-body F_ox_ kinetics was performed. After a 10-min warm-up at 20% 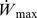, the power output was increased by 7.5% 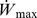 every 5-min until RER was >1.0 during the last minute of the stage.

### Indirect Calorimetry and Calculations

Oxygen uptake (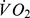), carbon dioxide output (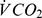) and ventilation (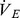) were measured continuously using a breath-by-breath system (Oxycon Pro, Jaeger, Würzburg, Germany). Before each test the gas analyzers were calibrated with gases of known concentration (16.00% O_2_ and 5.02% CO_2_), and the volume was automatically calibrated at two different flow rates (0.2 L⋅s^−1^ and 2 L⋅s^−1^). The HR was recorded continuously using an HR monitor (S810i, Polar Electro OY, Kempele, Finland).

During Test 1 and Test 2, HR and gas exchange data (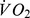,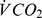) collected during the final 5-min of the pre-exercise resting phase and during the last 2-min of each stage of the submaximal incremental exercise test were averaged and used for calculations. RER was calculated as the ratio between 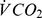 and 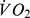, while F_ox_ and CHO_ox_ were calculated using stoichiometric equations [7], with the assumption that the urinary nitrogen excretion rate was negligible:
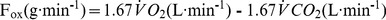
(1)

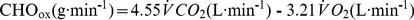
(2)


(1-RER) was also calculated given that the equation to calculate F_ox_ can be simplified to:
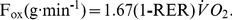
(3)


F_ox_ as a function of exercise intensity is reflected by two different linear relationships: a progressive decrease of (1–RER) and a linear increase of 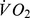 as power output is increased. The percentages of total energy expenditure derived from fat (% ENE_fat_) and CHO (% ENE_CHO_) were calculated [22]:
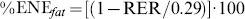
(4)

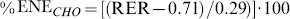
(5)


### Data Analysis Approaches to Determine Fat_max_

F_ox_ values obtained at each stage of the submaximal graded exercise test (which was terminated when RER was >1) were graphically depicted as a function of exercise intensity. Then, Fat_max_ and MFO (and subsequently RER, %HR_max_ at Fat_max,_ % 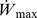 at Fat_max_) were determined using three different data analysis approaches (SIN, MV and P3). The “respiratory quotient technique” was not used in this study since it has been shown to be less accurate than the other methods [12].

#### SIN model

The SIN model [12] was used to model and characterize whole-body F_ox_ kinetics:
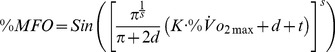
(6)


*Dilatation* (*d*), *symmetry* (*s)* and *translation* (*t*) are the three independent variables representing the main modulations of the curve. *K* is the constant of intensity and corresponds to (π/100). To fit the experimental data (*i.e.* F_ox_ rates) and to model the F_ox_ kinetics, the three variables were independently changed using an iterative procedure by minimizing the sum of the mean squares of the differences between the estimated energy derived from fat based on the SIN model and the energy derived from fat calculated from the raw F_ox_ data, as described in a previous study [12]. For each subject, Fat_max_ was calculated by differentiation of the SIN model equation. The Fat_max_ zone was determined as the range of exercise intensities with F_ox_ rates within 10% of MFO [3].

#### P3

Graphical depiction of F_ox_ values as a function of exercise intensity was performed by constructing a third polynomial curve with intersection at (0;0) [11]. Fat_max_ was calculated by differentiation of the P3 equation, and corresponded to the intensity at which the value of the differentiated equation was equal to zero.

#### Measured values

From the graphical representation of F_ox_ values as a function of exercise intensity, the stage at which the value of measured F_ox_ rates was maximal was determined, and the corresponding intensity was identified [3], [8]–[10], [23].

### Theoretical Example to Study how the CVs of 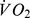 and 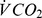 are Related to the CVs of RER and the CV of F_ox_

In order to investigate how the CV of 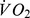 and 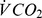 are linked to the CVs of parameters informing of substrate utilization (RER, F_ox_, CHO_fat_, 1-RER, ENE_fat_) three theoretical scenarios were created. 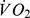 and 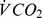
values for Test 1 and Test 2 were generated so that CVs of 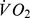 and 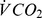 between Test 1 and Test 2 were ≤3%. A CV of ≤3% for 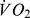 and 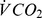 was chosen in line with results from previous studies [8], [18].

### Statistical Analysis

Data are expressed as the means ± standard deviation (SD) for all variables. Intra-individual CVs and LoA were calculated to test the variability between Test 1 and Test 2 for the following measures: a) Fat_max_ and physiological measures at Fat_max_ (MFO, RER, % HR_max_ and % 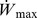) determined with three different data analysis approaches (SIN, MV and P3) and b) gas exchange data, HR and substrate oxidation rates at rest and during the first six stages of the submaximal incremental tests (from 20% to 57.5% of 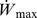). Intra-individual CVs were calculated for the physiological variables studied in the three theoretical scenarios.

Two-factorial analysis of variance for repeated measures (RMANOVA) was carried out to test for systematic changes in: a) Fat_max_, and physiological measures at Fat_max_ (factor 1: tests, factor 2: data analysis approaches), and b) gas exchange data, HR and substrate oxidation rates (factor 1: tests; factor 2: exercise intensity). For the same outcome measures, one-way RMANOVA was carried out to test for systematic changes in the intra-individual CV at Fat_max_.

Bland-Altman scatterplots are presented for Fat_max_ and MFO determined with SIN, P3 and MV. They show the difference between two corresponding measurements plotted against the mean of the measurements. Reference lines for the mean difference±1.96 SD are given. For all statistical analyses, the level of significance was set at *P*≤0.05. Statistical analysis was performed with the software SPSS 17.0 for Windows (SPSS, Chicago, IL) and Graph Pad Prism version 5.0 for Mac (GraphPad Software, San Diego, CA).

## Results

### Fat_max_ and Physiological Measures at Fat_max_ Determined with SIN, P3 and MV

Fat_max_ and physiological measures at Fat_max_ determined with three data analysis approaches (SIN, P3 and MV) are presented in Table 2. For all parameters, average values (n = 15) obtained from Test 1 and Test 2 were not significantly different (i.e. for Fat_max_: *P* = 0.37 for factor test and *P* = 0.20 for factor interaction between test and approach), indicating that no habituation or training effects occurred between testing sessions. Average values for Fat_max_ and related measures obtained with the three different approaches were also not significantly different (*i.e.* for Fat_max_: *P* = 0.13 for factor approach).

**Table 2:** Average values, limits of agreement and CVs for Fat_max_ and physiological measures at Fat_max_ determined with three approaches: SIN, P3 and MV.

		*SIN*	*P3*	*MV*
Fat_max_	Test 1	46.9±9.0	44.2±10.2	45.7±9.0
(% 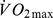 )	Test 2	48.9±12.2	48.6±13.1	49.6±12.6
	LoA	−29.7, 25.7	−36.7, 28.0	−32.0, 24.0
	CV (%)	16.4	20.8	18.6
MFO	Test 1	0.28±0.08	0.28±0.08	0.29±0.08
(g⋅min^−1^)	Test 2	0.29±0.13	0.29±0.13	0.30±0.12
	LoA	−0.27, 0.24	−0.25, 0.23	−0.26, 0.26
	CV (%)	25.3	22.8	26
RER Fat_max_	Test 1	0.91±0.02	0.91±0.02	0.91±0.02
	Test 2	0.91±0.02	0.91±0.02	0.91±0.02
	LoA	−0.05, 0.04	−0.06, 0.04	−0.06, 0.05
	CV (%)	1.6	1.7	1.6
%HR_max_ Fat_max_	Test 1	60.9±8.3	58.7±9.3	58.8±8.9
	Test 2	63.0±10.0	62.7±10.5	63.3±11.0
	LoA	−23.9, 19.7	−30.0, 22.2	−29.4, 20.4
	CV (%)	10	12.8*	12.8*
% 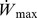 Fat_max_	Test 1	34.9±8.9	32.4±10.4	39.0±10.6
	Test 2	36.7±11.8	36.3±12.8	32.0±11.7
	LoA	−26.4, 22.6	−33.4, 25.6	−18.1, 32.1
	CV (%)	19.8	26.4*	24.9*

Values are means ± SD. LoA, limits of agreement; CV, coefficient of variation; SIN, sine model; MV, measured values; P3, 3^rd^ polynomial curve; Fat_max_, exercise intensity at which maximal fat oxidation rate occurs; MFO, maximal fat oxidation rate; RER Fat_max_, respiratory exchange ratio at Fat_max_; % HR_max_ Fat_max_, % maximal heart rate at Fat_max_; %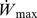 Fat_max_, % maximal aerobic power output at Fat_max_.

*P<0.05 between SIN and the other approaches (P3 and MV).

On the other hand, the within-individual CVs for Fat_max_ determined with SIN was 16.4% and tended to be lower (*P* = 0.10) than with P3 and MV (20.8% and 18.6% respectively). Similarly, the intra-individual CVs of % HR at Fat_max_ and % 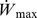 at Fat_max_ determined with SIN were significantly lower than with the other approaches (*P* = 0.043 and *P* = 0.05, respectively).

**Figure 1:**
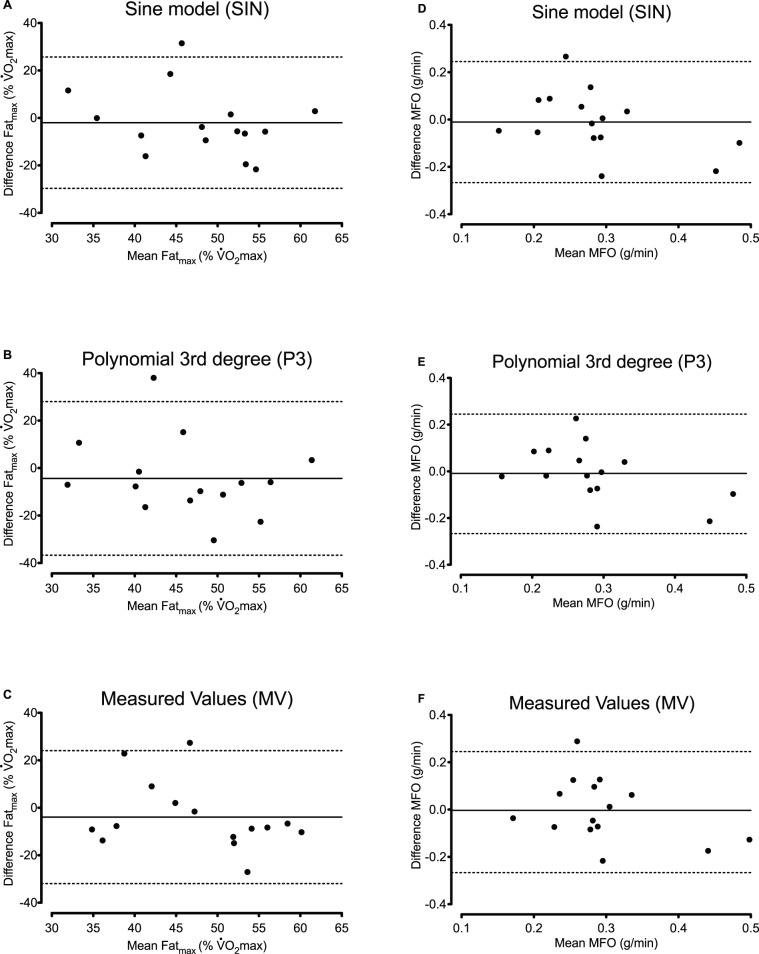
Bland-Altman plots of Fat_max_ and MFO determined with SIN, P3 or MV. SIN, sine model. P3, polynomial 3^rd^ degree; MV, measured values; Fat_max_, exercise intensity at which maximal fat oxidation rate occurs; 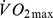, maximal oxygen uptake; MFO, maximal fat oxidation rate; Biases (*solid lines*) and 95% limits agreement (*dashed lines*).

The Bland–Altman scatterplots for Fat_max_ and MFO (Figure 1) reveal considerable intra-individual variability. The LoA for Fat_max_ were −2±27% of 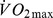 with SIN, −4±32% with P3, and −4±28% with MV. For MFO they were −0.01±0.25 g/min with SIN, 0.01±0.24 g/min with P3, and 0±0.26 g/min with MV (Table 2). A large between-individual difference in the variability between Test 1 and Test 2 was also seen. Accordingly, the CV at Fat_max_ ranged from 0 to 48%. For seven subjects it was under 10%, for two subjects it was between 10 and 15%, while for six subjects it was over 20%. However, the size of the difference between Test 1 and Test 2 appeared to be independent of the average value between the two measurements.

The difference in the HR at Fat_max_ between test 1 and 2 was <10 bpm in six participants, between 10 and 25 bpm in eight participants and was >25 bpm in one. In both tests, the range of HR frequencies corresponding to the Fat_max_ zone was broad (it was 38±8 bpm, and ranged from 95±16 to 133±20 bpm).

### Physiological Measures at Each Stage of a Submaximal Graded Test

**Figure 2:**
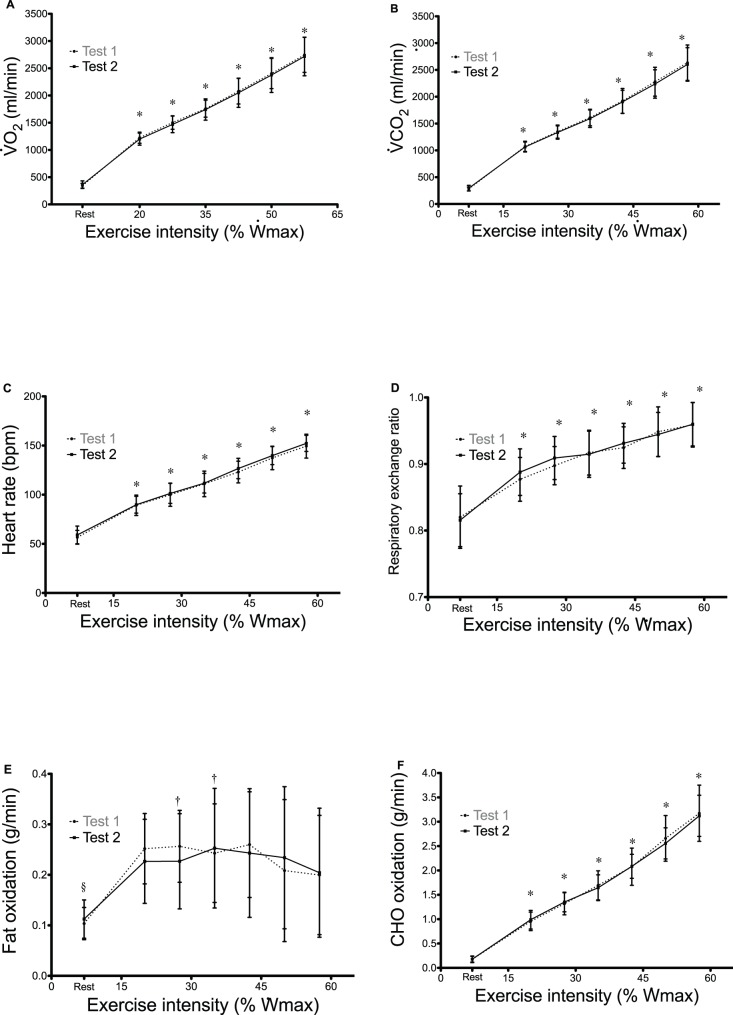
Course of average 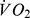, 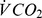, HR, RER, F_ox_ and CHO_ox_ during two identical submaximal incremental tests (mean and SD). 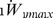
, maximal aerobic power output; 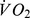, oxygen uptake; 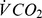, carbon dioxide production; RER, respiratory exchange ratio; HR, heart rate; F_ox_, fat oxidation rate; CHO_ox_, carbohydrate oxidation rate. *significantly increases with exercise intensity, §rest significantly different than exercise (20**–**57.5% 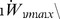), † significantly different than 57.5% 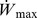.

The course of average 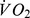,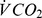, RER, HR, F_ox_ and CHO_ox_ in response to two identical submaximal graded test performed on separate days (Test 1 and Test 2) is presented in Figure 2. There was no significant difference between Test 1 and Test 2 in any of the parameters assessed. Average values of most physiological variables (except F_ox_) significantly increased with exercise intensity (*P*<0.001).

CVs of 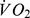,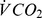, HR and RER were <10% at rest and <5% during exercise (Table 3). For instance, CVs for 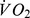,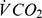 and RER were 7.5%, 9.1% and 3.8% at rest and were, on average, 3.1%, 3.0% and 2.5% during exercise. In contrast, CVs for CHO_ox_ and F_ox_ were markedly higher. The CV for F_ox_ was 20.6% at rest and ranged from 24.1 to 49.2% during exercise, while for CHO_ox_, it was 33.5% at rest and ranged from 8.5% to 12.9% during exercise. Interestingly, although the CV of RER was <4% under each condition, the CV of 1-RER was markedly higher (>20%), and was equal to the CV of the % of ENE_fat_. LoA between Test 1 and Test 2 are presented in Table 4.

**Table 3:** Coefficients of variation (%) for respiratory values and substrate oxidation rates in reponse to a submaximal graded exercise test.

	Rest	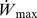 20%	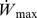 27.5%	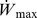 35%	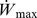 42.5%	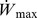 50%	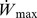 57.5%
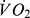 (ml⋅min^−1^)	7.5	4.0	3.0	2.9	3.3	3.1	2.6
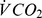 (ml⋅min^−1^)	9.1	3.4	3.1	3.0	2.5	3.0	3.0
HR (bpm)	5.7	3.7	4.2	4.4	3.6	2.6	2.5
RER	3.8	2.8	2.9	2.6	2.6	2.5	2.1
Fat_ox_ (g⋅min^−1^)	20.6	24.1	29.5	32	38.1	49.2	45.1
CHO_ox_ (g⋅min^−1^)	33.5	12.9	12.1	10.9	9.3	9.1	8.5
(1-RER)	20.6	20.9	24.1	28.0	30.8	36.6	47.9
ENE_fat_ (%)	20.6	20.9	24.1	28.0	30.8	36.6	47.9

Values are means. 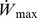, maximal aerobic power output; 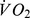, oxygen uptake; 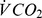, carbon dioxide production; HR, heart rate; RER, respiratory exchange ratio; Fat_ox_, fat oxidation rate; CHO_ox_, carbohydrate oxidation rate; ENE_fat_, % energy expenditure derived from fat.

**Table 4:** Limits of agreement between Test 1 and Test 2 for respiratory values and substrate oxidation rates in response to a submaximal graded exercise test.

	Rest	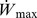 20%	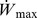 27.5%	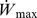 35%	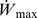 42.5%	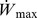 50%	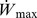 57.5%
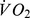 (ml⋅min^−1^)	−142,102	−139, 191	−117, 185	−146, 178	−206, 263	−241, 298	−225, 283
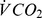 (ml⋅min^−1^)	−118, 89	−132, 155	−118, 151	−134, 178	−150, 186	188, 274	−236, 301
HR (bpm)	−16, 11	−11, 10	−15, 12	−19, 17	−18, 10	−16, 11	−20, 14
RER	−0.11, 0.12	−0.10, 0.07	−0.10, 0.07	−0.09, 0.10	−0.09, 0.08	−0.09, 0.10	−0.09, 0.09
Fat_ox_ (g⋅min^−1^)	−0.09, 0.07	−0.16, 0.21	−0.19, 0.25	−0.28, 0.26	−0.29, 0.33	−0.37, 0.32	−0.31, 0.30
CHO_ox_ (g⋅min^−1^)	−0.18, 0.18	−0.47, 0.40	−0.55, 0.48	−0.60, 0.69	−0.67, 0.65	−0.75, 0.95	−0.88, 0.99

Lower and higher limit of agreement are separated by a comma. 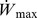, maximal aerobic power output; 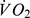, oxygen uptake; 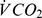, carbon dioxide production; HR, heart rate; RER, respiratory exchange ratio; Fat_ox_, fat oxidation rate; CHO_ox_, carbohydrate oxidation rate.

### Theoretical Example to Study how the CVs of 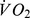 and 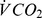 are Related to the CVs of RER and the CV of F_ox_

Three theoretical scenarios in which the CVs of 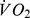 and 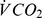 were ≤3% are presented in Table 5 and additional results with mathematical explanations are presented in appendix S1. In case scenario 1 and 2, the CVs of F_ox_ from were markedly different (3.1% *vs.* 38.2%) despite the CVs of 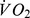 and 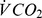 being identical (3%) (see Appendix S1, eq. 7, 8 and 9). Further, the CV of 1-RER was higher than the CV of RER, and was equal to the CV of %ENE_fat_. This difference was particularly apparent in case 2, where the CV of RER was 6%, while the CVs of F_ox_ and 1-RER were 38.2 and 35.3%, respectively.

**Table 5:** Three case scenario in which CV for 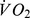 and 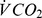 are≤3%.

	Case 1			Case 2			Case 3		
	Test 1	Test 2	CV (%)	Test 1	Test 2	CV (%)	Test 1	Test 2	CV (%)
**<b> 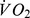 </b>**(ml⋅min^−1^)	**1699**	**1628**	**3.0**	**1699**	**1628**	**3.0**	**1699**	**1699**	**0.0**
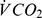 (ml⋅min^−1^)	**1450**	**1390**	**3.0**	**1390**	**1450**	**3.0**	**1390**	**1450**	**3.0**
RER	0.85	0.85	0.0	0.82	0.89	6.0	0.82	0.85	3.0
Fat_ox_ (g⋅min^−1^)	0.42	0.40	3.1	0.52	0.30	38.2	0.52	0.42	15.3
CHO_ox_ (g⋅min^−1^)	1.15	1.10	3.0	0.87	1.37	31.7	0.87	1.15	19.2
1-RER	0.15	0.15	0.1	0.18	0.11	35.3	0.18	0.15	15.3
% ENE_fat_	50.4	50.4	0.0	62.7	37.6	35.3	62.7	50.4	15.3

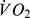
 and 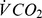, the values generated for the purpose of this study, are presented in bold. RER, Fatox, CHOox, 1-RER and % ENEfat were calculated. Case 1: CVs of 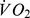 and 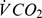 are 3% and the correlation coefficient between 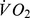 and 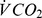 is positive; case 2: CVs of 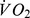 and 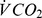 are 3% and the correlation coefficient between 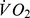 and 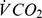 is negative; case 3: CV 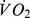 is 0% and CV 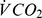 is 3%. When assuming CV 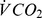 0% and CV 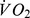 3%, similar results as for case 3 are obtained (data not shown). 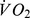, oxygen uptake; 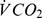, carbon dioxide production; RER, respiratory exchange ratio; Fatox, fat oxidation rate; CHOox, carbohydrate oxidation rate; % ENEfat, % energy derived from fat.

From the analysis of the three theoretical scenarios (as well as from the analysis of the whole dataset of 15 participants) we also observed that the CV of F_ox_ can be calculated from sum or subtraction of the CV of (1-RER) and the CV of 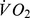 (Appendix S1, eq. 10 and 11). For example, in case 2, the CV of F_ox_ was 38.2% and was the sum of the CVs of 1-RER (35.3%) and 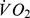 (3.0%). In case 3, the CV of F_ox_ was 15.3%, and equaled the CV of 1-RER (15.3%) ± CV 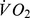 (0.0%).

## Discussion

In this study we assessed the reproducibility of Fat_max_ measurements determined with three different data analysis approaches and of CHO_ox_ and F_ox_ at rest (while sitting) and in response to each stage of an individualized graded test. We observed that the intra-individual variability of Fat_max_ was large (CV>16%) regardless of the data analysis approach employed and that F_ox_ at rest and at each stage of a graded test was also variable (CV>20%), despite the CVs of 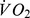,
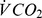
and RER being <5%.

The reproducibility of F_ox_ values at each stage of a graded test, despite being a key aspect in the determination of Fat_max_, was previously unexplored. In the current study, the CVs found for the parameters from which Fat_ox_ is calculated (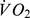,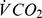 and RER) were in line with previous observations. At rest, the CV for RER was 3.8%, which closely mirrors the CV of 3.5% found by Roffey *et al.* [17]. In the present study the resting assessment was performed with the individuals in a seated position and this needs to be taken into consideration when making comparisons with studies in which resting metabolism was assessed with participants lying supine. During exercise, the average CVs for 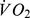,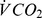 and RER were 3.1%, 3.0% and 2.5%, respectively, and were similar or lower than those reported in previous investigations [8], [13], [18], [19]. Despite this, the CVs found for F_ox_ were >20%. This shows that even though CHO_ox_ and F_ox_ are calculated from 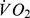 and 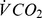 by means of the stoichiometric equations [7], a low variability in those parameters is not necessarily indicative of low variability in CHO_ox_ and F_ox_.

To further study how the CVs of 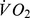 and 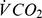 are related to the CVs of RER and the CV of F_ox_, three theoretical scenarios were created. At present, scientific reports as well as companies validating calorimeters tend to draw information on the variability of substrate oxidation from the CVs of 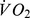, 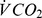 and RER. The results of the theoretical scenarios (Table 5) and the mathematical explanations presented in the appendix S1 illustrate that those CVs do not provide sufficient information on the variability of substrate oxidation rates.

As can be seen in case 2, when the 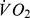 and 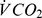 vary in different directions between two tests (increase in 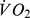 and decrease in 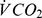 or *viceversa*), the variability of F_ox_ is high. This is because in such conditions, the standard deviation of F_ox_ results from the sum of the standard deviations of 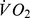 and 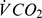, multiplied by a factor 1.67. Therefore, in addition to the size of the change in 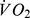 and 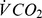 between tests, it is crucial to know whether they change in the same or opposite sense between measurements.

The RER is the ratio between 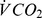 and 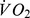 and, therefore, provides information on the relationship between those measurements. However, in the theoretical scenarios the CV of RER remains low (<6%) also when the variability in F_ox_ is high (>30%), showing that the CV of RER is not a parameter adequately informing on the variability in the proportion of nutrients utilized. This is because the RER is value bounded in an interval separate from zero (0.7–1.0) and therefore the CV is not an adequate measure to assess the variability of RER. On the other hand, the CV of 1-RER appears to be an informative marker on the variability in F_ox_ rates: it provides the same results as the CV of ENE_fat_, it accounts for a large proportion of the CV of F_ox_, and it is simple to calculate.

In this study, as well as in other studies investigating the reproducibility of indirect calorimetry measures [8], [13], [14], [16]–[19], the total variation observed between Test 1 and Test 2 is the sum of both biological and equipment variation. It was beyond the scope of this study to assess the relative contribution of each. However, the average variation of the equipment (gas analysis system) used in this study is known. It was assessed using a portable metabolic simulator (which excludes any biological variability) and the average CV for 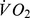 and 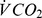 was 1.9±0.6% and 1.3±0.5% respectively [18].

In addition to investigating the variability in F_ox_ and related parameters at each stage of a graded test, a novel feature of this study was the assessment of the intra-individual variability in Fat_max_ determined with the SIN model and its comparison with the variability of Fat_max_ measures obtained using different data analysis approaches. All the approaches to determine Fat_max_ presented in the literature were compared in this analysis, except the “respiratory quotient technique”, since it has previously been shown to be less accurate [12]. The comparison revealed that the intra-individual CV at Fat_max_ was higher than 16% with any of the data analysis approaches employed and that there was a relatively small difference between approaches. However, the CVs of Fat_max_, % 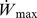 at Fat_max_ and of % HR_max_ determined with SIN were lower than with P3 and MV, possibly because the SIN model provides an accurate and more complete description of the F_ox_ kinetics as a function of exercise intensity than the other data analysis approaches. These results support the use of SIN over other approaches in future studies given that it is more reliable and provides more detailed information.

The intra-individual variability of Fat_max_ and related parameters found in this study was in line with those of Meyer *et al.* [14]. In the present study the LoA for Fat_max_ determined with SIN were −2.0±27.7 of 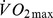, while in the study from Meyer *et al.* LoA for Fat_max_ of −3.9±27.7 of 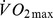 were observed. Further, also consistent with the results published by Meyer *et al.* [14], the within-individual variability was markedly different between individuals. On the other hand, the CV for Fat_max_ observed in this study, on average, was slightly higher than those reported in other studies [8], [13], [24]. The lower CV found by Achten *et al.* [8] (9.6%) could be due to the fact that measurements were repeated three times [and the CV generally decreases when the number of measurements increases [25]] and were performed in trained athletes, who may have a less variable response to exercise than individuals with a lower training level. Overall, the differences in the results obtained between studies are difficult to interpret, particularly because most studies only report average results, and do not present “individual responses” and/or ranges. This highlights the need for a better understanding of the determinants of intra-individual variability in Fat_max._

Previous studies in the field considered an intra-individual variability of ±10 bpm in the HR at Fat_max_ acceptable, since this value reflects a realistic margin in individuals who use HR for the monitoring of training intensity [8], [14]. In the present study this target was met by the majority, but not all, participants. However, the range of intensities at which F_ox_ is within 10% of MFO (Fat_max_ zone) was broad and this was consistent with previous observations [3], [26]. Therefore, despite its variability, training prescription at Fat_max_ ensures that high rates of F_ox_ are elicited on different days.

The determination of F_ox_ and Fat_max_ (and therefore the determination of their variability) is influenced by a number of methodological factors including the exercise test design, the data analysis approach and the pre-test conditions. In this study, a robust methodological approach was employed. The submaximal graded exercise was individualized based upon the results of a maximal test. It started at 20% of 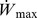 and the workload was subsequently increased by 7.5% 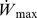 every 5-min. This ensured the reaching of a steady state [27] and allowed to study F_ox_ at several intensities (participants performed at least six exercise stages with an RER<1). Further, the statistical analysis was carried out in accordance with the recommendations for reliability assessment in sport medicine [15].

Pre-test conditions included a 10-hour overnight fast and 24 hours of standardization in diet and physical activity prior to each submaximal graded exercise test. This level of standardization was adopted because it appears to be the most commonly employed approach in our research field [3], [8], [28]–[32] and because more rigorous standardization is difficult to achieve both in out-clinic and research settings. Despite the standardization adopted, in some individuals a high intra-individual variability in Fat_max_ and related variables was found, suggesting that a longer period of standardization (≥2 days prior to testing) might be needed to improve the reproducibility of those measures. However, while more strict pre-test standardization leads to greater internal validity, it also leads to poorer external validity (i.e harder translation of the results into practice). More generally, while the validity of using a graded exercise tests to determine Fat_max_ has been reported in a number of studies [3], [13], [33], [34], a recent study questions the usefulness using this approach to prescribe training in populations such as highly trained athletes [35]”.

A number of questions on the reproducibility of substrate metabolism during exercise are still to be answered. Further research is required to: a) describe how standardization in physical activity and diet prior to testing impact on reliability of measurements, b) study the determinants of the variability in CHO_ox_ and F_ox_ and c) explore the reproducibility in F_ox_ in other cohorts including overweight and untrained individuals.

In summary, we have shown here that the intra-individual variability in Fat_max_ is high (CV>16%) and is highly variable between individuals, regardless of the data analysis approach employed. The intra-individual variability at rest and in response to an individualized graded test is high for F_ox_ measures (CV>20% for F_ox_) although it is low for 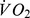, 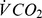 and RER (CV<5%). The CV of (1-RER) appears to be a more representative measure of the variability in substrate oxidation than CV of RER. Training prescription at Fat_max_ can be useful clinically given that, despite its variability, it results in Fat_ox_ rates within 90% of MFO on different days. In a research setting, differences in Fat_max_ and F_ox_ within and between groups can be detected as long as a sufficiently large number of participants is recruited. Further research in this area is required.

## Supporting Information

Appendix S1 (DOCX)
