# Correction: Reproducibility of Fat_max_ and Fat Oxidation Rates during Exercise in Recreationally Trained Males

**DOI:** 10.1371/journal.pone.0114115

**Published:** 2014-11-17

**Authors:** The PLOS ONE Staff

The third, fourth, and fifth authors’ names are spelled incorrectly. The correct names are: Nuala M Byrne, Rachel E Wood, and Ingrid J Hickman. The correct citation is: Croci I, Borrani F, Byrne NM, Wood RE, Hickman IJ, et al. (2014) Reproducibility of Fat_max_ and Fat Oxidation Rates during Exercise in Recreationally Trained Males. PLoS ONE 9(6): e97930. doi:10.1371/journal.pone.0097930

Table 2 has been corrected for improved readability. Please see the corrected Table 2 here.

**Table 2:** Average values, limits of agreement and CVs for Fatmax and physiological measures at Fatmax determined with three approaches: SIN, P3 and MV.

		*SIN*	*P3*	*MV*
Fat_max_ (% 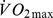 )	Test 1	46.9 ± 9.0	44.2 ±10.2	45.7 ± 9.0
	Test 2	48.9 ± 12.2	48.6 ±13.1	49.6 ± 12.6
	LoA	-29.7, 25.7	-36.7, 28.0	-32.0, 24.0
	CV (%)	16.4	20.8	18.6
MFO (g⋅min^-1^)	Test 1	0.28 ±0.08	0.28 ± 0.08	0.29 ± 0.08
	Test 2	0.29 ±0.13	0.29 ± 0.13	0.30 ± 0.12
	LoA	-0.27, 0.24	-0.25, 0.23	-0.26, 0.26
	CV (%)	25.3	22.8	26
RER Fat_max_	Test 1	0.91 ± 0.02	0.91 ± 0.02	0.91 ± 0.02
	Test 2	0.91 ±0.02	0.91 ± 0.02	0.91 ± 0.02
	LoA	-0.05, 0.04	-0.06, 0.04	-0.06, 0.05
	CV (%)	1.6	1.7	1.6
%HR_max_ Fat_max_	Test 1	60.9 ± 8.3	58.7 ± 9.3	58.8 ± 8.9
	Test 2	63.0 ± 10.0	62.7 ± 10.5	63.3 ± 11.0
	LoA	-23.9, 19.7	-30.0, 22.2	-29.4, 20.4
	CV (%)	10	12.8 *	12.8*
% 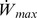 Fat_max_	Test 1	34.9 ± 8.9	32.4 ± 10.4	39.0 ± 10.6
	Test 2	36.7 ± 11.8	36.3 ± 12.8	32.0 ± 11.7
	LoA	-26.4, 22.6	-33.4, 25.6	-18.1, 32.1
	CV (%)	19.8	26.4 *	24.9*

Values are means ± SD. LoA, limits of agreement; CV, coefficient of variation; SIN, sine model; MV, measured values; P3, 3^rd^ polynomial curve; Fat_max_, exercise intensity at which maximal fat oxidation rate occurs; MFO, maximal fat oxidation rate; RER Fat_max_, respiratory exchange ratio at Fat_max_; % HR_max_ Fat_max_, % maximal heart rate at Fat_max_; %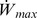 Fat_max_, % maximal aerobic power output at Fat_max_. * P < 0.05 between SIN and the other approaches (P3 and MV).

Table 3 contains multiple errors. Please see the corrected Table 3 here.

**Table 3:** Coefficients of variation (%) for respiratory values and substrate oxidation rates in reponse to a submaximal graded exercise test.

	Rest	20 % 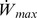	27.5% 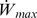	35 % 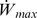	42.5% 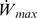	50% 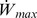	57.5% 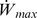
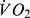 (ml⋅min^-1^)	7.5	4.0	3.0	2.9	3.3	3.1	2.6
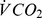 (ml⋅min^-1^)	9.1	3.4	3.1	3.0	2.5	3.0	3.0
HR (bpm)	5.7	3.7	4.2	4.4	3.6	2.6	2.5
RER	3.8	2.8	2.9	2.6	2.6	2.5	2.1
Fat_ox_ (g⋅min^-1^)	20.6	24.1	29.5	32	38.1	49.2	45.1
CHO_ox_ (g⋅min^-1^)	33.5	12.9	12.1	10.9	9.3	9.1	8.5
(1-RER)	20.6	20.9	24.1	28.0	30.8	36.6	47.9
ENE_fat_ (%)	20.6	20.9	24.1	28.0	30.8	36.6	47.9

Values are means. 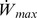, maximal aerobic power output; 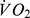, oxygen uptake; 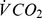, carbone dioxide production; HR, heart rate; RER, respiratory exchange ratio; Fat_ox_, fat oxidation rate; CHO_ox_, carbohydrate oxidation rate; ENE_fat_, % energy expenditure derived from fat.

Table 4 contains multiple errors. Please see the corrected Table 4 here.

**Table 4:** Limits of agreement between Test 1 and Test 2 for respiratory values and substrate oxidation rates in response to a submaximal graded exercise test.

	Rest		20 % 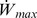	27.5 % 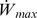	35 % 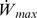	42.5% 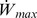	50% 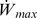	57.5% 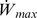
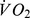 (ml⋅min^-1^)		-142,102	-139, 191	-117, 185	-146, 178	-206, 263	-241, 298	-225, 283
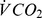 (ml⋅min^-1^)		-118, 89	-132, 155	-118, 151	-134, 178	-150, 186	188, 274	-236, 301
HR (bpm)		-16, 11	-11, 10	-15, 12	-19, 17	-18, 10	-16, 11	-20, 14
RER		-0.11, 0.12	-0.10, 0.07	-0.10, 0.07	-0.09, 0.10	-0.09, 0.08	-0.09, 0.10	-0.09, 0.09
Fat_ox_ (g⋅min^-1^)		-0.09, 0.07	-0.16, 0.21	-0.19, 0.25	-0.28, 0.26	-0.29, 0.33	-0.37, 0.32	-0.31, 0.30
CHO_ox_ (g⋅min^-1^)		-0.18, 0.18	-0.47, 0.40	-0.55, 0.48	-0.60, 0.69	-0.67, 0.65	-0.75, 0.95	-0.88, 0.99

Lower and higher limit of agreement are separated by a comma. 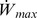, maximal aerobic power output; 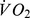, oxygen uptake; 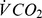, carbone dioxide production; HR, heart rate; RER, respiratory exchange ratio; Fat_ox_, fat oxidation rate; CHO_ox_, carbohydrate oxidation rate.

Table 5 contains multiple errors. Please see the corrected Table 5 here.

**Table 5:** Three case scenario in which CV for 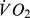 and 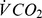 are ≤ 3%.

	Case 1			Case 2			Case 3		
	Test 1	Test 2	CV (%)	Test 1	Test 2	CV (%)	Test 1	Test 2	CV (%)
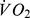 (ml⋅min^-1^)	**1699**	**1628**	**3.0**	**1699**	**1628**	**3.0**	**1699**	**1699**	**0.0**
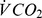 (ml⋅min^-1^)	**1450**	**1390**	**3.0**	**1390**	**1450**	**3.0**	**1390**	**1450**	**3.0**
RER	0.85	0.85	0.0	0.82	0.89	6.0	0.82	0.85	3.0
Fat_ox_ (g⋅min^-1^)	0.42	0.40	3.1	0.52	0.30	38.2	0.52	0.42	15.3
CHO_ox_ (g⋅min^-1^)	1.15	1.10	3.0	0.87	1.37	31.7	0.87	1.15	19.2
1-RER	0.15	0.15	0.1	0.18	0.11	35.3	0.18	0.15	15.3
% ENE_fat_	50.4	50.4	0.0	62.7	37.6	35.3	62.7	50.4	15.3

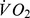
 and 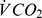, the values generated for the purpose of this study, are presented in bold. RER, Fatox, CHOox, 1-RER and % ENEfat were calculated. Case 1: CVs of 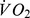 and 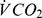 are 3% and the correlation coefficient between 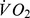 and 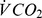 is positive; case 2: CVs of 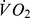 and 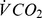 are 3% and the correlation coefficient between 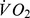 and 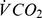 is negative; case 3: CV 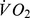 is 0% and CV 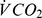 is 3%. When assuming CV 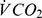 0% and CV 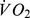 3%, similar results as for case 3 are obtained (data not shown). 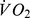, oxygen uptake; 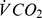, carbon dioxide production; RER, respiratory exchange ratio; Fatox, fat oxidation rate; CHOox, carbohydrate oxidation rate; % ENEfat, % energy derived from fat.

There are a number of errors in the legend for Figure 2. Please see the complete, corrected Figure 2 here.

**Figure 2:**
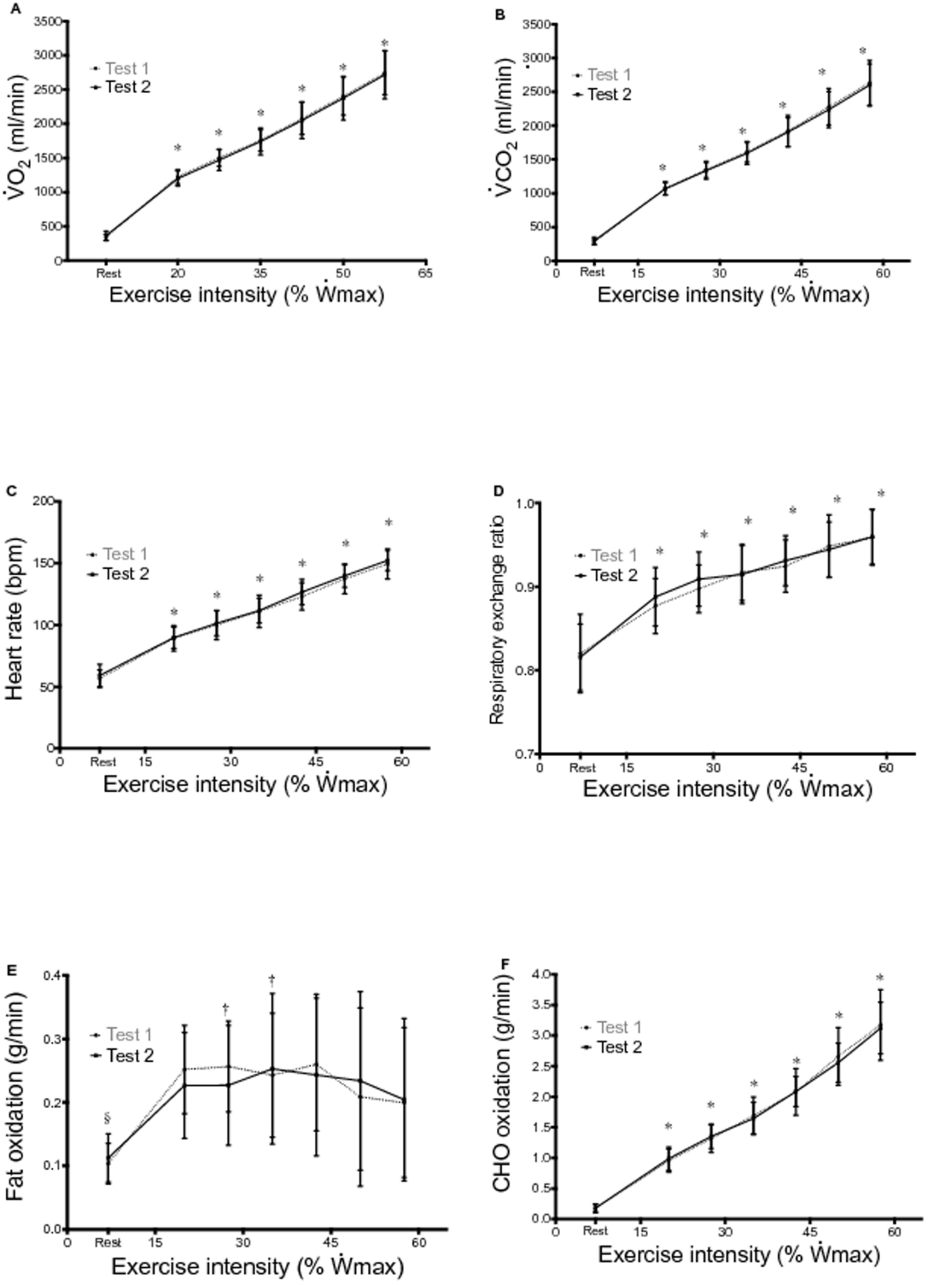
Course of average 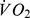, 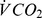, HR, RER, F_ox_ and CHO_ox_ during two identical submaximal incremental tests (mean and SD). 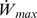
, maximal aerobic power output; 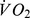, oxygen uptake; 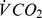, carbon dioxide production; RER, respiratory exchange ratio; HR, heart rate; F_ox_, fat oxidation rate; CHO_ox_, carbohydrate oxidation rate. * significantly increases with exercise intensity, § rest significantly different than exercise (20-57.5% 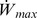), † significantly different than 57.5% 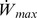.
