# Supplementary material for: Reproducibility of Fatmax and Fat Oxidation Rates during Exercise in Recreationally Trained Males
Source: PLoS One. 2014 Jun 2;9(6):e97930. doi: 10.1371/journal.pone.0097930 (PMC4041727; doi:10.1371/journal.pone.0097930)
Supplement: Appendix S1 — (DOCX) [file pone.0097930.s001.docx]

**Appendix**

***Theoretical example to study how the CVs of*** $\dot{V}O_{2}$ ***and***$\dot{V}CO_{2}$***are related to the CVs of RER and F_ox_***

$\dot{V}O_{2}$ and $\dot{V}CO_{2}$values for Test1 and Test2 were generated. In case 1 and 2, the CVs of $\dot{V}O_{2}$ and $\dot{V}CO_{2}$ were identical (3%), while the correlation coefficient (*r*) between $\dot{V}O_{2}$ and $\dot{V}CO_{2}$ $\left[r\text{(}\dot{V}O_{2},\dot{V}CO_{2}\text{)}\right]$ was different. It was positive in case 1 because both $\dot{V}O_{2}$ and $\dot{V}CO_{2}$ were lower in Test2 compared to Test1, while it was negative in case 2 because $\dot{V}O_{2}$ and $\dot{V}CO_{2}$ changed in opposite directions; in case 3 the CV of $\dot{V}O_{2}$was 0% and the CV of $\dot{V}CO_{2}$ was 3%.

***Links between the CVs of*** $\dot{V}O_{2}$*,*$\dot{V}CO_{2}$ ***and RER and the CV of F_ox_***

The CVs of F_ox_ from case scenario 1 and 2 were markedly different, despite the CVs of $\dot{V}O_{2}$ and $\dot{V}CO_{2}$ being identical (3%). This difference is explained by the different $r\text{(}\dot{V}O_{2},\dot{V}CO_{2}\text{)}$ assumed for case 1 and 2, highlighting that $r\text{(}\dot{V}O_{2},\dot{V}CO_{2}\text{)}$ importantly influences the standard deviation of F_ox_ which is calculated:

Eq.7

where $\sigma$is the standard deviation and *r* the coefficient of correlation.

This equation, when data from only 2 tests are used, can be simplified:

Eq.8

when $r\text{(}\dot{V}O_{2},\dot{V}CO_{2}\text{)}$ is negative, and

Eq.9

when $r\text{(}\dot{V}O_{2},\dot{V}CO_{2}\text{)}$ is positive.

From the analysis of the three theoretical scenarios as well as from the analysis of the whole dataset (15 participants), we observed that the CV of F_ox_ is related to the CV of (1-RER) and the CV of $\dot{V}O_{2}$ as follows:

Eq.10

when $r\text{(}1-RER,\dot{V}O_{2}\text{)}$ is positive, and

Eq.11

when $r\text{(}1-RER,\dot{V}O_{2}\text{)}$ is negative.
